# Delayed Start of Routine Vaccination in Preterm and Small-for-Gestational-Age Infants: An Area-Based Cohort Study from the Tuscany Region, Italy

**DOI:** 10.3390/vaccines10091414

**Published:** 2022-08-28

**Authors:** Vieri Lastrucci, Monia Puglia, Martina Pacifici, Primo Buscemi, Michela Sica, Giorgia Alderotti, Gilda Belli, Elettra Berti, Franca Rusconi, Fabio Voller

**Affiliations:** 1 Epidemiology Unit, Meyer Children’s Hospital, Viale Gaetano Pieraccini 24, 50139 Florence, Italy; 2 Observatory of Epidemiology, Regional Health Agency of Tuscany, Via Pietro Dazzi, 1, 50141 Florence, Italy; 3 Medical Specialization School of Hygiene and Preventive Medicine, University of Florence, Viale GB Morgagni 48, 50134 Florence, Italy; 4 Neonatology and Neonatal Intensive Care Unit, Azienda Sanitaria Locale Toscana Centro, Piazza Santa Maria Nuova, 1, 50122 Firenze, Italy; 5 Neonatal Intensive Care Unit, Meyer Children’s Hospital, Viale Gaetano Pieraccini 24, 50139 Florence, Italy; 6 Department of Mother and Child Health, Azienda USL Toscana Nord Ovest, Via Cocchi 7/9, 56121 Pisa, Italy

**Keywords:** preterm infants, full-term infants, small for gestational age, routine vaccinations, vaccination, immunization, timeliness, delay, area-based cohort, full birth cohort

## Abstract

Preterm and small-for-gestational-age (SGA) infants are more susceptible to vaccine-preventable diseases. To evaluate routine vaccination timeliness in these high-risk groups, a full birth cohort of infants (*n* = 41,502) born in 2017 and 2018 in Tuscany was retrospectively followed up until 24 months of age. Infants were classified by gestational age (GA) and SGA status. The vaccinations included: hexavalent (HEXA), measles-mumps-rubella, varicella, pneumococcal conjugate (PCV), and meningococcal C conjugate. Time-to-event (Kaplan–Meier) analyses were conducted to evaluate the timing of vaccination according to GA; logistic models were performed to evaluate the associations between GA and SGA with vaccination timeliness. Time-to-event analyses show that the rate of delayed vaccine receipt increased with decreasing GA for all the vaccinations, with a less marked gradient in later vaccine doses. Compared to full-term infants, very preterm infants significantly showed an increased odds ratio (OR) for delayed vaccination in all the vaccinations, while moderate/late preterm infants only showed an increased OR for HEXA-1, HEXA-3, PCV-1, and PCV-3. SGA infants had a significantly higher risk of delayed vaccination only for HEXA-1 and PCV-1 compared to non-SGA infants. In conclusion, vaccinations among preterm and SGA infants showed considerable delay. Tailored public health programs to improve vaccination timeliness are required in these high-risk groups.

## 1. Introduction

Timely immunization is essential to protect infants from vaccine-preventable diseases; a delayed immunization leads to an increased period of susceptibility and thus to an increased infection risk. Preterm infants (PTI) are more susceptible to infections compared to those born full term due to immunological immaturity and the decreased transfer of maternal antibodies, which are mainly transferred during the last trimester of pregnancy [1,2]. Therefore, PTI are more exposed to the more severe consequences of vaccine-preventable diseases. A history of prematurity is associated with severe pertussis infections, and PTI are at increased risk of invasive pneumococcal diseases compared to full-term infants (FTI) [2,3,4,5,6]. For these reasons, timely vaccination is even more important for PTI.

Several studies have shown that routine immunization schemes based on chronological age are both safe and effective in developing protective immune responses in preterm and low-birth-weight infants [7,8,9]. To ensure early protection, PTI who are otherwise healthy should be vaccinated following the same schedule used for FTI. According to international guidelines on vaccination, the schedule for PTI should be based on their chronological age rather than corrected gestational age (GA) and regardless of their birth weight [10,11,12]. 

Despite these recommendations, a delayed onset of immunizations and under-vaccination were observed in PTI, low-birth-weight (birthweight less than 2500 g) and small-for-gestational-age (SGA) infants [4,13,14,15,16,17,18,19,20,21,22,23,24]. A delayed start of immunization was observed in 39.5% of all PTI in the Netherlands, and a study conducted in Washington State showed that over half of PTI were under-vaccinated at 19 months [13,19]. Furthermore, PTI who are also SGA—commonly defined as a weight below the 10th percentile for the gestational age [25]—seem to be at higher risk of delayed vaccination compared to PTI with a normal birth weight for the corresponding GA, thus suggesting that both GA and birth weight have an independent effect on the timeliness of immunization in PTI [16]. 

Although the rate of preterm births is increasing in most Western countries [26], there are limited studies assessing the timeliness of routine vaccinations in this high-risk group. In particular, most of the studies had a limited sample size, data collected from the self-reporting of parents or from the records of a specific health care provider that cannot track vaccinations which occurred elsewhere [13,15,20,22,23,24]. Only a few studies are based on a large cohort, and even fewer studies were carried out in area-based, full birth cohorts which have the advantage of minimizing selection bias, depicting the whole population and providing a natural control group of FTI [16,19,21]. Furthermore, it is still unclear whether the immunization delay of PTI registered in the first doses persists in the administration of subsequent doses in the series. 

In Italy, routine immunizations are carried out through a network of public vaccination clinics, and childhood vaccinations recommended in the Italian national Immunization program are actively offered and guaranteed free of charge for all children [27]; as for the timing of vaccine administration, it is recommended not to correct for GA or birth weight [28]. At the time of the study, the following vaccines were actively offered in the first two years of life, starting from the third month of age: hexavalent (HEXA, including diphtheria, tetanus, pertussis, Haemophilus influenzae type b, hepatitis B, and inactivated polio), measles-mumps-rubella (MMR), measles-mumps-rubella-varicella (MMRV), varicella (Var), conjugate pneumococcal (PCV), conjugate meningococcal C (MenC), and meningococcal B (MenB) [26]. 

The aim of this study was to evaluate—in a full birth cohort of infants born in 2017 and 2018 in the Tuscany region, Italy—the timeliness of the routine immunization schedule in different categories of preterm infants and SGA infants compared to infants born at term and of adequate weight for GA. Furthermore, a secondary aim of the study was to explore factors associated with delayed vaccinations.

## 2. Materials and Methods

The study was conducted in accordance with the Declaration of Helsinki and approved by the Ethics Committee of the Tuscany region (Comitato Etico Regionale per la Sperimentazione Clinica della Regione Toscana, Sezione: comitato etico pediatrico) (protocol code 183/2020 and 5 August 2020).

### 2.1. Study Design, Population, and Data Collection

This is a retrospective cohort study conducted on the administrative data of the Public Health Care System (TPHCS) of the Tuscany region. Tuscany is a region with a population of more than 3.7 million residents located in central Italy. The TPHCS provides universal health coverage for all Tuscany residents, and routine immunizations are offered free of charge to all infants according to the Italian routine vaccination schedule.

All infants born in Tuscany from 1 January 2017 to 31 December 2018 who were registered in the Tuscany region birth registry were included in the study. The data of infants on vaccination and residence were linked from the Tuscany region vaccination registry and from the Tuscany region population registry using an anonymous unique identifier. Infants were followed up until 24 months of age. Infants with a missing/erroneous unique identifier, those with missing data for GA, and those who died or changed region of residence during the follow-up period were excluded. Furthermore, for each one of the vaccines considered, infants who resulted in being completely unvaccinated at the end of the follow-up were excluded because the study aimed to evaluate determinants of timeliness of vaccinations and not those associated with no vaccination [16]. There were no differences in the proportions of completely unvaccinated infants by GA or SGA status: considering all the groups, this proportion ranged between 2.5 and 3.2%.

### 2.2. Outcomes and Covariates

The primary outcome was the timeliness of vaccination. The following vaccines were considered: HEXA, MMRV, MMR, V, PCV, and MenC. For each vaccine, the timing of the doses foreseen by the regional routine vaccination schedule were considered [29]. In particular, the following vaccine doses and timings were considered:

- HEXA vaccine: first dose (between 61st and 90th day) and third dose (between 306th and 395th day);
- MMR vaccine (included MMR or MMRV vaccination): first dose (between 396th and 455th day);
- Var vaccine (included Var or MPRV vaccination): first dose (between 396th and 455th day);
- PCV vaccine: first dose (between 61st and 90th day) and third dose (between 396th and 455th day);
- MenC vaccine: first dose (between 366th and 455th day).

For each participant, the timing of each vaccination was calculated based on chronological age, without correction for GA. Doses administered a day after the recommended period were considered as delayed. Furthermore, vaccination delay was further characterized considering the following categories: minimal delay (delayed less than one month), moderate delay (delayed 1–3 months), severe delay (delayed 3–6 months), and extreme delay (delayed 6 months or more).

Furthermore, the timeliness of the first vaccinations and of vaccine doses to be administered by the 15th month of life was also studied. Administration a day after the 90th day of at least one between HEXA-1 and PCV-1 vaccines was defined as delayed vaccination at first vaccination; similarly, administration a day after the 455th day of at least one among HEXA-3, MMR, V, PCV-3, and MenC vaccines was considered delayed vaccination at the 15th month of life. 

Maternal data and the data of infants were retrieved from the Tuscany region birth registry. For each infant the following covariates were considered: date of birth; sex; GA at birth; SGA status (<10th percentile, ≥10th percentile); multiple births; sibling birth order (first-born/only child, having older siblings); conception by assisted reproductive technologies (ART); urbanization level of residence (urban areas, rural areas); and birth hospital level of newborn care (first level—low-risk care, second level—special care /neonatal intensive care unit). SGA status was calculated according to the Italian reference charts (considering birthweight, GA, parity and sex) [30]. Further details concerning the urbanization level of residence classification are reported in the Supplementary Materials (Appendix 1).

As far as the maternal data are concerned, the following variables were considered: age at delivery (<25 years, 25–34 years, >34 years); nationality (Italian, foreign nationality); education level (less than high school diploma, high school diploma or higher); and employment status (unemployed, employed).

### 2.3. Statistical Analysis

Categorical variables were presented as percentages, while continuous variables were described as median and interquartile range (IQR). In the descriptive analysis, infants were stratified based on GA at birth into four groups: very preterm infants (VPTI: GA < 32 weeks), moderate and late preterm infants (MLPTI: GA between 32 and 36 weeks), full-term infants (FTI: GA ≥ 37 weeks).

For each one of the considered vaccine doses, a time-to-event analysis using the Kaplan–Meier method was calculated to evaluate the timing of vaccination according to GA; log-rank test was performed to compare the survival curves of the different GA groups (<28 weeks, 28–31 weeks, 32–36 weeks, ≥37 weeks).

A multivariate logistic regression analysis was performed to evaluate the associations between GA and SGA status with vaccination timeliness. Multivariate logistic regression models were fitted for each one of the considered vaccine doses. In the models, the GA variable was categorized into three groups (VPTI, MLPTI, and FTI); the SGA status variable was categorized into two groups (<10th percentile, ≥10th percentile). All the models included the following independent variables: sex, multiple birth, sibling birth order, conception by ART, urbanization level of residence, birth hospital level of newborn care, maternal age at delivery, nationality, maternal education level, and maternal employment status.

In order to evaluate determinants of delayed vaccination, two distinct logistic regression models were performed considering the outcomes of timeliness of the first vaccinations and of vaccine doses to be administered by the 15th month of life. The following independent variables were considered: GA, SGA, sex, multiple birth, sibling birth order, conception by ART, urbanization level of residence, birth hospital level of newborn care, maternal age at delivery, nationality, maternal education level, and maternal employment status.

Data analysis was performed using Stata 15 SE (StataCorp LP, College Station, TX, USA).

## 3. Results

A total of 52,164 infants were born in Tuscany from 1 January 2017 to 31 December 2018. From these, 11,112 (21.3%) infants were excluded from the study: 591 (1.1%) infants had a missing or erroneous unique identifier, 8793 (16.8%) infants died or did not reside in the Tuscany region during the follow-up period, and 1270 (2.4%) infants resulted in being completely unvaccinated at the end of the follow-up. A total of 41,502 (79.6%) infants were included in the study. In the studied population, 20,204 infants were females, representing around the 49% of the sample (see Table 1). A total of 3158 (7.6%) infants were born preterm: specifically, 2802 (6.7%) were MLPTI, and 356 (0.9%) were VPTI. A total of 3990 (9.6%) infants were born SGA. Infants of multiple births were 1590 (3.8%), and those conceived by ART were 1729 (4.2%) (see Table 1). As for other infants and maternal variables, these are presented in Table 1.

Data on age at vaccination and timeliness of vaccination by GA and SGA status are reported in Table 2 and Table 3, respectively. Considering the whole population, the proportion of infants vaccinated on time was over 75% for the HEXA first dose, MenC, and PCV first dose. The lowest proportion of infants vaccinated on time was observed for the MMR (41.1%) and Var (38.2%) vaccines. A higher median age at immunization was found in preterm infants compared to FTI for all the considered vaccine doses. A trend in the age at vaccination according to GA was observed for all the considered vaccines: as gestational age increases, the mean age at vaccination decreases. The proportion of infants vaccinated on time was lower in preterm infants compared to FTI in all the considered vaccines. The highest differences in the proportion of on time vaccinations were observed for the HEXA first dose (69.3% of preterm infants vs. 77.4% of FTI) and PCV first doses (68.9% of preterm infants vs. 77.8% of FTI). For all the considered vaccines, the proportion of infants vaccinated on time increased with GA. In particular, on time administration of the first doses of HEXA and PCV occurred in 39.7% and 38.4% of VPTI, 72.2% and 72% of MLPTI, and in 77.4% and 77.8% of FTI, respectively. Across GA classes, the lowest differences in the percentage of on time vaccination were observed for MenC and PCV third dose administration (MenC: 73% of VPTI vs. 81.8% of FTI; PCV: 54.4% of VPTI vs. 62.4% of FTI). As for SGA status, the proportion of infants vaccinated on time was similar between SGA and non-SGA infants in all the considered vaccine doses, except for the first doses of HEXA and PCV in which the proportion of infants vaccinated on time was lower in SGA infants.

**Figure 1:**
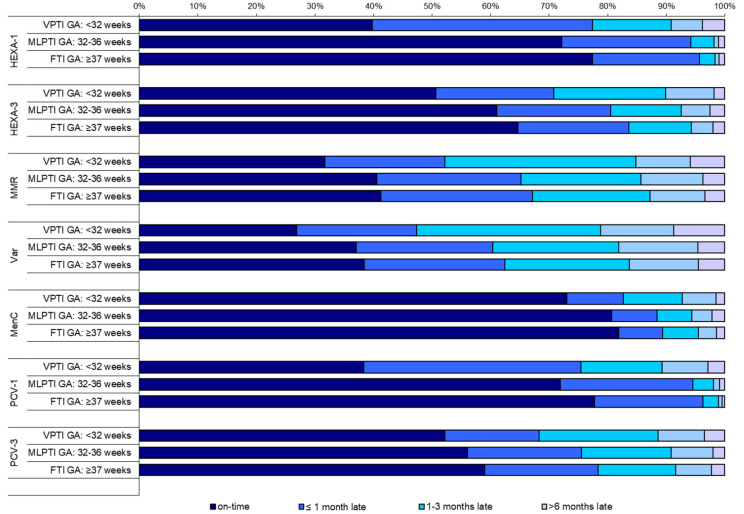
Distribution of vaccination timing (on time, ≤1 month late, between 1 and 3 months late, ≥6 months late) by prematurity classes (very preterm infants—VPTI; moderate and late preterm infants—MLPTI; full-term infants—FTI) for HEXA-1, HEXA-3, MMR, Var, MenC, PCV-1, and PCV-3 vaccine doses.

The distribution of vaccination timing for GA and vaccine doses is reported in Figure 1. Considering the group of infants who had a delayed vaccination, a minimal delay represented the highest proportion of MLPTI and FTI in all the vaccine doses; as for VPTI, a moderate delay represented the highest proportion of infants with delayed vaccination for MMR, Var and MenC vaccines. The proportion of infants with an extreme delay at vaccination was higher in preterm infants in all the vaccine doses; in particular, VPTI registered the higher proportion of extreme delay in all the vaccines, except for HEXA-3. MenC. Var and MMR vaccines showed the highest proportion of extreme delay both in VPTI (Var 8.7%; MMR 5.9%) and MLPTI (Var 4.6%; MMR 3.7%).

**Figure 2:**
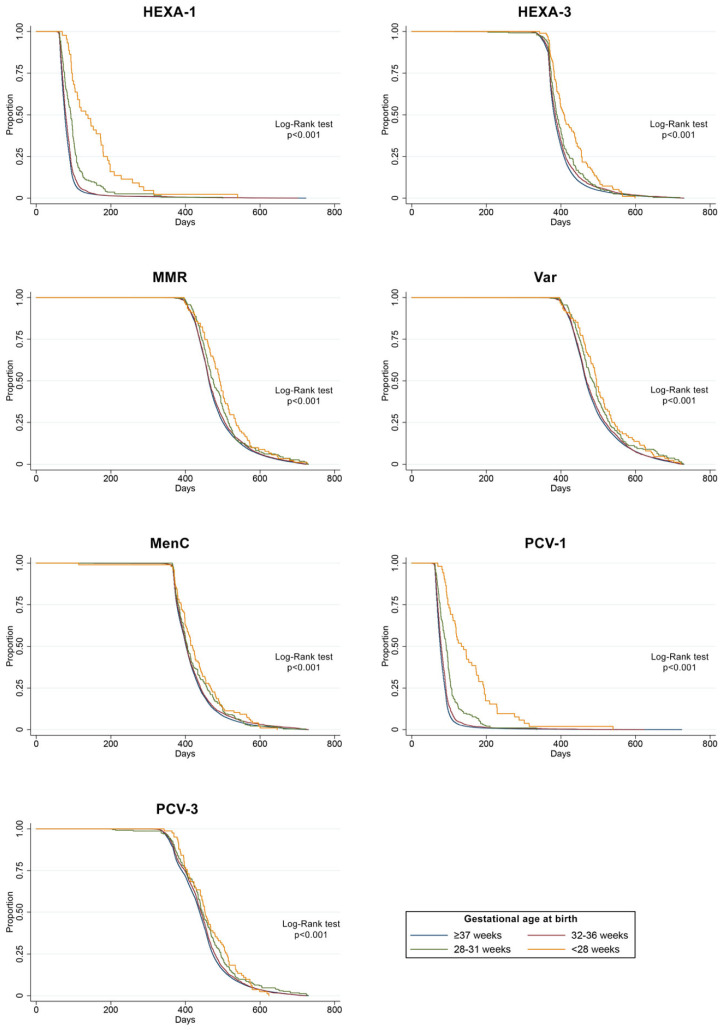
Kaplan–Meier curves for time to start of immunization by gestational age at birth (<28 weeks, 28–31 weeks, 32–36 weeks, ≥37 weeks) for HEXA-1, HEXA-3, MMR, Var, MenC, PCV-1, and PCV-3 vaccine doses.

Figure 2 shows the Kaplan–Meier curves for time at the start of immunization by gestational age. Time-to event analyses showed that the immunization start was delayed with decreasing GA for all the vaccinations with the log-rank test showing a significant difference in the onset of vaccination in groups with different gestational ages (*p* < 0.001). Infants born before 28 weeks and those born between 28 and 32 weeks of gestation showed the highest rate of delayed vaccine receipt in all the vaccine doses. The differences across GA groups in vaccination onset were less marked in later vaccine doses (e.g., HEXA-3 and PCV-3).

**Figure 3:**
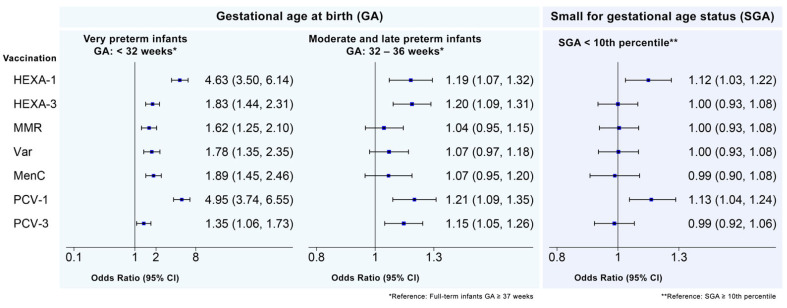
Forest plot reporting adjusted odds ratios (OR) of vaccination delay for preterm infants (compared to full-term infants) and SGA status (compared to infants who were not born SGA) for HEXA-1, HEXA-3, MMR, Var, MenC, PCV-1, and PCV-3 vaccine doses. * Reference: full-term infants GA ≥ 37 weeks; ** Reference: SGA ≥ 10th percentile.

In Figure 3, the adjusted odds ratios of vaccination delay for GA and SGA groups for each vaccine considered are summarized. The full results of each multivariate logistic regression model performed are reported in the Supplementary Materials (see Tables S1–S7). Compared to FTI, VPTI showed a significantly increased odds ratio of delayed vaccination for all vaccines considered; the highest strength of the association was registered for the first doses of HEXA and PCV (OR 4.63 and OR 4.95, respectively). MLPTI showed a significantly increased odds ratio of delayed vaccination compared to FTI for the first and third doses of HEXA (OR 1.19 and OR 1.20, respectively) and PCV (OR 1.21 and OR 1.15) (Figure 3). As for SGA status, infants who were SGA—compared to infants who were not SGA—showed a significantly increased odds ratio of delayed vaccination only in the first doses of HEXA and PCV (OR 1.12 and OR 1.13, respectively).

The results of the multivariate logistic regression models evaluating the determinants of delayed vaccination for the first vaccinations and for vaccine doses to be administered by 15th month of life are reported in Table 4. Infants of multiple births showed an increased odds ratio of delayed vaccination both for first vaccinations and for vaccine doses to be administered by the 15th month of life. Conversely, infants who were first born /only child showed a significantly lower risk of delayed vaccination in both models. In addition, infants conceived by ART showed a decreased odds ratio of delayed vaccination both for first vaccinations and for vaccine doses to be administered by the 15th month of life, although this association did not reach statistical significance (*p* = 0.07) for first vaccinations. Maternal employment status and age at delivery were not significantly associated with delayed vaccination in both models. A number of other factors did not act consistently between the two models. In particular, infants born in second level hospitals and infants living in rural areas showed a significantly increased risk of delayed first vaccinations. Maternal foreign nationality was significantly associated with a lower risk of delayed vaccination only for vaccinations to be administered by the 15th month of age. A lower maternal education level was significantly associated with a decreased risk of vaccination delay for first vaccinations, while it was associated with an increased risk of delay for vaccinations to be administered by the 15th month of age.

## 4. Discussion

The present study assessed the timeliness of immunization in an area-based, full birth cohort of the Tuscany region, Italy with the aim of comparing immunization timeliness between PTI and FTI as well as between infants born SGA and those who were not SGA. In total, around 70% of MLPTI and 40% of VPTI received their first immunizations on time compared to more than 77% of FTI. We showed that PTI were at higher risk of delayed vaccination compared to FTI in all the routine vaccinations considered; in particular, this increased risk of delayed vaccination was present in all prematurity classes, with VPTI presenting the highest risk of delay. Furthermore, the entity of the increased risk of delayed vaccination in PTI was higher in vaccine doses administered early in life, such as HEXA and PCV first doses, compared to later vaccine doses such as HEXA and PCV third doses, and MMR, Var, and MenC. As far as SGA status is concerned, we found that these infants had a significantly increased risk of delayed vaccination for the first doses of HEXA and PCV compared to infants who were not SGA; contrary to what we observed in PTI, such increased risk for SGA was not present in all the later vaccine doses considered. Multivariate regression analyses show that multiple births negatively influenced the timeliness of the first immunizations and of those to be administered by the 15th month, while infants who were first born/only child and those conceived by ART showed a timelier administration of these vaccines. 

To the best of our knowledge, this is the first study assessing the timeliness of routine immunizations in a full birth cohort in Italy. Many years ago, a study was conducted in an Italian cohort composed of only very preterm infants; furthermore, in this study, the vaccination timeliness was evaluated in absolute terms, and no comparisons with FTI or with other preterm categories were made to evaluate the delay in relation to other reference groups [14]. 

Despite international guidelines and recommendations indicating that PTI should be vaccinated following the same schedule used for FTI [10,11,12], a vaccination delay has already been observed in PTI [16,19,21]. Vaccination delay increases the risk of vaccine-preventable diseases, and PTI are more vulnerable to infections as a consequence of immunological immaturity and the decreased transfer of maternal antibodies [1,2,3,4,5,6]. Possible explanations for vaccination delay in this high-risk group may be related to concerns of inadequate immune response to vaccines and fear about a higher susceptibility to adverse vaccine events. Indeed, although vaccines are proven to be safe and effective in developing an adequate immune response in PTI [7,8,9], an inadequate level of knowledge and adherence to international guidelines for immunization of PTI has been reported among health professionals [31]. In light of this, the finding that vaccine doses that have to be administered earlier in life were those presenting the highest risk of delayed immunization may indicate that these concerns and misconceptions wane—at least in part—over time. 

Independently from gestational age at birth, infants who were born SGA were at a higher risk of delayed vaccination only for the doses of first vaccines—i.e., HEXA-1 and PCV-1. The role of SGA in influencing the timeliness of immunization is scarcely investigated in the literature. Similar to our findings, Woesteberg et al. [16] reported that SGA status was associated with a delayed vaccination for the first dose of the DTaP-IPV vaccine in PTI. However, this study did not consider later vaccine doses within the series or other vaccinations administered at older ages. Vaccination concerns and beliefs similar to those reported for preterm infants may be the basis of this delayed vaccination in SGA infants.

First-born infants and infants conceived by ART experienced a timelier vaccination both at the first vaccine doses and for vaccine doses foreseen in later ages. These findings are probably related to the fact that—in both cases—there may be increased attention from parents with regards to all the aspects related to the health of their child, including vaccinations. On the other hand, multiple birth infants showed a higher risk of delayed vaccination, irrespective of their gestational age, and this may be explained by the higher organizational difficulties in attending vaccination appointments experienced by parents of multiple birth infants. 

Maternal sociodemographic variables examined in our study did not show a clear influence on the timeliness of vaccination. While maternal age and employment status were not associated with the timeliness of immunization, maternal education level and nationality were associated with the timeliness of immunization for vaccines doses to be administered in later ages. In particular, a higher education level and a foreign nationality were associated with a timelier vaccination. As for the maternal education level, a higher level of vaccine literacy and better opportunities for obtaining information probably explain the finding. As for the role of nationality, it is possible to speculate that foreigners may tend to rely more on official information provided by the primary care and preventive care services, while Italians may tend to have multiple sources of information, ultimately resulting in increased risk of exposure to misinformation about vaccine safety and benefits, especially for vaccines that are more strongly linked to false claims such as the MMR vaccine [32]. 

The present study has several strengths. In addition to being carried out on a very large cohort of infants, the study involved an area-based, full birth cohort, thus providing an accurate picture of the timeliness of vaccination in the Tuscany region, Italy and minimizing potential selection bias. The selection of a full birth cohort has also allowed natural comparison groups—i.e., FTI and infants who were not born SGA—to evaluate the vaccination timeliness in preterm and SGA infants not only in absolute terms but also in relative terms. Furthermore, the study is based on data registered in the birth and vaccination regional registries; these data are directly and punctually collected by obstetrics, nurses, and doctors who provided birth care (within 10 days after birth) or administered the vaccines, respectively. These data collection procedures minimize the risk of misclassifications and recall bias. Lastly, this is one of the first studies that evaluated vaccination timeliness in SGA and non-SGA infants.

The study also has some limitations. Firstly, a proportion of infants registered in the birth registry were not linked with the vaccination registry and thus were excluded from the study. This was due to multiple causes: infant death after birth, infants who moved to another region during the follow-up period, and inherent technical problems in record linkage. However, this exclusion is probably not related to the characteristics of infants and vaccination timeliness and likely biased results towards the null. Indeed, the characteristics of the population studied and reported in Table 1 are superimposable in terms of prevalence to those of the whole population of infants born in Tuscany in the same period. In particular, the prevalence of VPTI and MLPTI in the birth registries were exactly the same as the ones reported by our sample [33]. Secondly, while interpreting the results of the study, it should be taken into account that unvaccinated infants were excluded from the study since the study aimed to study factors associated with the timeliness of vaccinations and not factors associated with no vaccination; this may have the potential to alter the interpretation of the results if the proportion of unvaccinated infants differed across the different groups. However, in our study, the number of unvaccinated children was low, and there was no significant difference in the proportion of unvaccinated infants in the GA and SGA groups, thus this exclusion likely had no or little role in altering the interpretation of the results in our study. Thirdly, the evaluation of factors influencing the timeliness of vaccination was limited to variables available in the registries considered. Although, various and varied factors were considered, it was not possible to explore the role of other factors reported to be associated with the timeliness of immunization, such as paternal socioeconomic and demographic characteristics, the level of health literacy of parents, and healthcare provider factors [14,34,35]. Furthermore, we did not have data concerning the health conditions of infants that may have influenced the timelines of vaccination. 

## 5. Conclusions

This large area-based, full birth cohort study shows that preterm infants—including MLPTI—had a significant delay in first immunizations compared to FTI. Although to a lesser degree, the timeliness gap between FTI and PTI persisted in later vaccine doses within the series and in vaccine doses that have to be administered at older ages. The findings of the study highlight an increased risk of vaccine-preventable infections in a population group that is already exposed to a greater vulnerability to infections. Several parental and provider factors may explain vaccination delay in PTI, including vaccine safety and efficacy beliefs, perceptions of medical vulnerability, knowledge and understanding of vaccine recommendations, as well as vaccine communication between doctors and families. Further research is needed to better identify specific reasons behind vaccination delay in PTI. Timeliness of immunization in PTI remains a public health priority and strategies that integrate multiple interventions, such as training of health professionals, tailored parental information and education programs, and improved accessibility to immunization services are needed.

## Figures and Tables

**Table 1:** Characteristics of the study population for the total sample: prematurity classes (very preterm infants—VPTI; moderate and late preterm infants—MLPTI; full-term infants—FTI) and SGA status at birth (<10th percentile; ≥10th percentile).

	Total Sample	Gestational Age at Birth			SGA Status	
	N (%) 41,502	VPTI GA: <32 Weeks N (%)	MLPTI GA: 32–36 Weeks N (%)	FTI GA: ≥37 Weeks N (%)	<10th Percentile N (%)	≥10th Percentile N (%)
**Sex**						
Male	21,295 (51.3)	191 (53.6)	1489 (53.1)	19,615 (51.2)	2134 (53.5)	19,103 (51.1)
Female	20,204 (48.7)	165 (46.4)	1313 (46.9)	18,726 (48.8)	1856 (46.5)	18,300 (48.9)
**Gestational age at birth**						
FTI GA: ≥37 weeks	38,344 (92.4)				3609 (9.4)	34,637 (90.6)
MLPTI GA: 32–36 weeks	2802 (6.7)				350 (12.5)	2443 (87.5)
VPTI GA: <32 weeks	356 (0.9)				31 (8.8)	323 (91.2)
**SGA status**						
<10th percentile	3990 (9.6)	31 (8.8)	350 (12.5)	3609 (9.4)		
≥10th percentile	37,403 (90.4)	323 (91.2)	2443 (87.5)	34,637 (90.6)		
**Pregnancy outcome**						
Multiple	1590 (3.8)	115 (32.3)	897 (32.0)	578 (1.5)	339 (8.5)	1246 (3.3)
Singleton	39,912 (96.2)	241 (67.7)	1905 (68.0)	37,766 (98.5)	3651 (91.5)	36,157 (96.7)
**Conceived by ART ^1^**						
Yes	1729 (4.2)	70 (19.9)	401 (14.4)	1258 (3.3)	202 (5.1)	1521 (4.1)
No	39,571 (95.8)	281 (80.1)	2384 (85.6)	36,906 (96.7)	3772 (94.9)	35,700 (95.9)
**Urbanization level of residence**						
Urban area	30,297 (73.3)	270 (76.0)	2091 (75.0)	27,936 (73.2)	2869 (72,5)	27,352 (73.4)
Rural area	11,024 (26.7)	85 (24.0)	698 (25.0)	10,241 (26.8)	1089 (27.5)	9902 (26.6)
**Birth hospital level**						
First level	19,850 (47.8)	28 (7.9)	995 (35.5)	18,827 (49.1)	1840 (46.1)	17,922 (47.9)
Second level	21,649 (52.2)	328 (92.1)	1807 (64.5)	19,514 (50.9)	2150 (53.9)	19,478 (52.1)
**Maternal nationality**						
Italian	31,750 (76.7)	253 (71.1)	2141 (76.5)	29,356 (76.7)	3109 (78.1)	28,570 (76.5)
Foreign nationality	9667 (23.3)	103 (28.9)	658 (23.5)	8906 (23.3)	870 (21.9)	8776 (23.5)
**Maternal age at delivery**						
<25 years	3225 (7.8)	24 (6.7)	168 (6.0)	3033 (7.9)	309 (7.8)	2902 (7.8)
25–34 years	22,425 (54.0)	169 (47.5)	1296 (46.3)	20,960 (54.7)	2079 (52.1)	20,285 (54.2)
>34 years	15,844 (38.2)	163 (45.8)	1336 (47.7)	14,345 (37.4)	1601 (40.1)	14,209 (38.0)
**Maternal education level**						
High school diploma or higher	9763 (24.1)	88 (25.8)	681 (25.1)	8994 (24.0)	1058 (27.1)	8670 (23.7)
Less than high school diploma	30,800 (75.9)	253 (74.2)	2036 (74.9)	28,511 (76.0)	2841 (72.9)	27,901 (76.3)
**Maternal employment status**						
Employed	13,645 (34.0)	116 (36.0)	888 (33.3)	12,641 (34.0)	1380 (35.8)	12,232 (33.8)
Unemployed	26,500 (66.0)	206 (64.0)	1782 (66.7)	24,512 (66.0)	2470 (64.2)	23,960 (66.2)
**Sibling birth order**						
Having older sibling	20,088 (48.4)	131 (36.8)	1240 (44.3)	18,717 (48.9)	1895 (47.5)	18,160 (48.5)
First born/only child	21,370 (51.5)	225 (63.2)	1559 (55.7)	19,586 (51.1)	2905 (52.5)	19,243 (51.5)

^1^ ART = assisted reproductive technologies.

**Table 2:** Data on age at vaccination (median age and interquartile range—IQR) and timeliness of vaccination for prematurity classes (very preterm infants—VPTI; moderate and late preterm infants—MLPTI; full-term infants—FTI).

	HEXA-1 (N = 36,494)		HEXA-3 (N = 39,612)		MMR (N = 38,730)		Var (N = 37,989)		MenC (N = 38,977)		PCV-1 (N = 36,097)		PCV-3 (N = 38,360)	
	Median Age in Days (IQR)	N Vaccinated on Time (%)	Median Age in Days (IQR)	N Vaccinated on Time (%)	Median Age in Days (IQR)	N Vaccinated On Time (%)	Median Age in Days (IQR)	N Vaccinated on Time (%)	Median Age in Days (IQR)	N Vaccinated on Time (%)	Median Age in Days (IQR)	N Vaccinated on Time (%)	Median Age in Days (IQR)	N Vaccinated on Time (%)
**Gestational age**														
FTI GA: ≥37 weeks	77 (68–89)	26,146 (77.4)	382 (370–408)	23,693 (64.7)	463 (437–501)	14,776 (41.3)	467 (439–513.5)	13,506 (38.4)	402 (377–438)	29,476 (81.8)	76 (68–89)	25,967 (77.8)	438 (390–473)	22,128 (62.4)
MLPTI GA: 32–36 weeks	79 (70–92)	1787 (72.2)	385 (371–412)	1627 (61.1)	464 (438–505)	1055 (40.6)	469 (441–519)	942 (37.1)	403 (378–441)	2121 (80.7)	79 (70–92)	1771 (71.7)	442 (398–479)	1537 (59.5)
VPTI GA: <32 weeks	96 (77–116)	95 (39.7)	394 (374–437)	170 (50.6)	479.5 (447–519)	102 (31.7)	490 (452.5–532.5)	84 (26.9)	408.5 (383–460)	241 (73)	97 (78–119)	94 (38.4)	448 (402–498)	172 (54.4)
Total <37 weeks	80 (70–93)	1882 (69.3)	386 (371–414)	1797 (59.9)	465 (439–508)	1157 (39.6)	470 (442–522)	1026 (36)	404 (379–442)	2362 (79.9)	80 (70–93)	1865 (68.9)	443 (399–481)	1709 (58.9)
**Total**	**77 (68–89)**	**28,028 (76.8)**	**382 (370–409)**	**25,490 (64.3)**	**463 (438–501)**	**15,933 (41.1)**	**467 (440–514)**	**14,532 (38.2)**	**402 (377–438)**	**31,838 (81.7)**	**77 (68–89)**	**27,832 (77.1)**	**438 (391–474)**	**23,837 (62.1)**

**Table 3:** Data on age at vaccination (median age and interquartile range—IQR) and timeliness of vaccination for SGA status at birth (<10th percentile; ≥10th percentile) for HEXA-1, HEXA-3, MMR, Var, MenC, PCV-1, and PCV-3 vaccine doses.

	HEXA-1 (N = 36,390)		HEXA-3 (N = 39,506)		MMR (N = 38,627)		Var (N = 37,888)		Menc (N = 38,878)		PCV-1 (N = 35,995)		PCV-3 (N = 38,256)	
	Median Age in Days (IQR)	N Vaccinated on Time (%)	Median Age in Days (IQR)	N Vaccinated on Time (%)	Median Age in Days (IQR)	N Vaccinated on Time (%)	Median Age in Days (IQR)	N Vaccinated on Time (%)	Median Age in Days (IQR)	N Vaccinated on Time (%)	Median Age in Days (IQR)	N Vaccinated on Time (%)	Median Age in Days (IQR)	N Vaccinated on Time (%)
**SGA status**														
≥10th percentile	78 (68–89)	25,339 (77)	382 (370–409)	22,977 (64.3)	463 (438–501)	14,366 (41.1)	467 (440–514)	13,098 (38.2)	402 (377–438)	28,718 (81.7)	77 (68–89)	25,170 (77.3)	438 (391–474)	21,474 (62.1)
<10th percentile	77 (69–91)	2598 (74.4)	383 (370–408)	2435 (64.1)	463 (437–503)	1517 (40.9)	468 (439–515)	1386 (38)	403 (378–437)	3038 (81.6)	77 (69–91)	2571 (74.5)	438 (392–473)	2292 (62.1)
**Total**	**77 (68–89)**	**27,937 (76.8)**	**382 (370–409)**	**25,412 (64.3)**	**463 (438–501)**	**15,883 (41.1)**	**467 (440–514)**	**14,484 (38.2)**	**402 (377–438)**	**31,756 (81.7)**	**77 (68–89)**	**27,741 (77.1)**	**438 (391–474)**	**23,766 (62.1)**

**Table 4:** Multivariate logistic regression models: determinants of delayed vaccination for first vaccinations (HEXA-1 and PCV-1) and for vaccine doses to be administered by 15th month of life (MMR, Var, PCV-3, and MenC).

	First Vaccinations: HEXA-1 and PCV-1			Vaccine Doses to Be Administered by 15th Month of Life: MMR, Var, PCV-3 and MenC		
	OR	95%CI	*p*	OR	95%CI	*p*
**Gestational age at birth**						
FTI GA: ≥37 weeks	Ref.					
MLPTI GA: 32–36 weeks	1.20	1.08–1.34	<0.001	1.08	0.98–1.18	0.11
VPTI GA: <32 weeks	4.82	3.65–6.36	<0.001	1.64	1.26–2.13	<0.001
**SGA status**						
≥10th percentile	Ref.					
<10th percentile	1.13	1.04–1.23	0.004	1.01	0.94–1.08	0.78
**Sex**						
Male	Ref.					
Female	0.99	0.94–1.04	0.67	0.95	0.91–0.99	0.02
**Pregnancy outcome**						
Singleton	Ref.					
Multiple	1.31	1.14–1.51	<0.001	1.16	1.02–1.32	0.02
**Conceived by ART ^1^**						
No	Ref.					
Yes	0.88	0.77–1.01	0.07	0.80	0.72–0.90	<0.001
**Urbanization level of residence**						
Urban area	Ref.					
Rural area	1.10	1.04–1.16	0.001	0.94	0.9–0.99	0.02
**Birth hospital level**						
First level	Ref.					
Second level	1.39	1.32–1.46	<0.001	1.02	0.97–1.06	0.39
**Maternal nationality**						
Italian	Ref.					
Foreign nationality	1.03	0.96–1.10	0.38	0.77	0.73–0.81	<0.001
**Maternal age at delivery**						
<25 years	Ref.					
25–34 years	0.92	0.83–1.02	0.13	1.03	0.94–1.12	0.54
>34 years	1.06	0.95–1.18	0.3	1.03	0.94–1.13	0.49
**Maternal education level**						
High school diploma or higher	Ref.					
Less than high school diploma	0.93	0.87–0.99	0.03	1.08	1.03–1.14	0.002
**Maternal employment status**						
Employed	Ref.					
Unemployed	1.06	1.00–1.12	0.06	1.01	0.96–1.06	0.65
**Sibling birth order**						
Having older sibling	Ref.					
First born/ only child	0.75	0.72–0.80	<0.001	0.83	0.79–0.87	<0.001

^1^ ART = assisted reproductive technologies.

## Data Availability

The data presented in this study are available on request from the corresponding author. Data are not publicly available due to the current regulation on privacy.

## Supplementary Materials

The following supporting information can be downloaded at https://www.mdpi.com/article/10.3390/vaccines10091414/s1. Appendix 1: Classification of the urbanization level of residence [36]; Table S1a: Crude odd ratios for delayed vaccination for HEXA-1 for prematurity classes (very preterm infants—VPTI; moderate and late preterm infants—MLPTI; full-term infants—FTI) and SGA status; Table S1b: Adjusted odds ratios for delayed vaccination for HEXA-1 for prematurity classes (very preterm infants—VPTI; moderate and late preterm infants—MLPTI; full-term infants—FTI) and SGA status; Table S2a: Crude odds ratios for delayed vaccination for HEXA-3 for prematurity classes (very preterm infants—VPTI; moderate and late preterm infants—MLPTI; full-term infants—FTI) and SGA status; Table S2b: Adjusted odds ratios for delayed vaccination for HEXA-3 for prematurity classes (very preterm infants—VPTI; moderate and late preterm infants—MLPTI; full-term infants—FTI) and SGA status; Table S3a: Crude odds ratios for delayed vaccination for MMR for prematurity classes (very preterm infants—VPTI; moderate and late preterm infants—MLPTI; full-term infants—FTI) and SGA status; Table S3b: Adjusted odds ratios for delayed vaccination for MMR for prematurity classes (very preterm infants—VPTI; moderate and late preterm infants—MLPTI; full-term infants—FTI) and SGA status; Table S4a: Crude odds ratios for delayed vaccination for Var for prematurity classes (very preterm infants—VPTI; moderate and late preterm infants—MLPTI; full-term infants—FTI) and SGA status; Table S4b: Adjusted odds ratios for delayed vaccination for Var for prematurity classes (very preterm infants—VPTI; moderate and late preterm infants—MLPTI; full-term infants—FTI) and SGA status; Table S5a: Crude odds ratios for delayed vaccination for MenC for prematurity classes (very preterm infants—VPTI; moderate and late preterm infants—MLPTI; full-term infants—FTI) and SGA status; Table S5b: Adjusted odds ratios for delayed vaccination for MenC for prematurity classes (very preterm infants—VPTI; moderate and late preterm infants—MLPTI; full-term infants—FTI) and SGA status; Table S6a: Crude odds ratios for delayed vaccination for PCV-1 for prematurity classes (very preterm infants—VPTI; moderate and late preterm infants—MLPTI; full-term infants—FTI) and SGA status; Table S6b: Adjusted odds ratios for delayed vaccination for PCV-1 for prematurity classes (very preterm infants—VPTI; moderate and late preterm infants—MLPTI; full-term infants—FTI) and SGA status; Table S7a: Crude odds ratios for delayed vaccination for PCV-3 for prematurity classes (very preterm infants—VPTI; moderate and late preterm infants—MLPTI; full-term infants—FTI) and SGA status; Table S7b: Adjusted odds ratios for delayed vaccination for PCV-3 for prematurity classes (very preterm infants—VPTI; moderate and late preterm infants—MLPTI; full-term infants—FTI) and SGA status.
