# Supplementary material for: Delayed Start of Routine Vaccination in Preterm and Small-for-Gestational-Age Infants: An Area-Based Cohort Study from the Tuscany Region, Italy
Source: Vaccines (Basel). 2022 Aug 28;10(9):1414. doi: 10.3390/vaccines10091414 (PMC9503094; doi:10.3390/vaccines10091414)
Supplement: Supplementary file 1 [file vaccines-10-01414-s001.zip › vaccines-1829048-supplementary.pdf]

Appendix 1. Classification of the urbanization level of residence

Urbanization level of residence

The classification of the urbanization level of residence adopted in this study was developed by the Department for the Economic Development and Cohesion of the Ministry of economic development of Italy; this classification is currently adopted for policy purposes and statistical reporting at national and regional level [36].

The municipality of residence were used to classify the urbanization level and the following categories were adopted:

- Urban areas
- Suburban areas
- Rural areas
- Isolated rural areas

Urban areas were defined as the municipalities that simultaneously offers:

- At least one high school for each of the specialization foreseen by Italian education system (lyceum; technical institute; professional institute)
- At least one hospital with a type 1 emergency department (*i.e.* Italian health care system define a type 1 emergency department a 24-hour service with full resuscitation facilities provided of short-stay and cardiac intensive care units, shock room, multi-specialty emergency services and dedicated diagnostic imaging and laboratory services)
- At least one railway station of middle-small size with high volume of passengers ("Silver category" accordingly to the Italian railway network classification)

According to tertiles of the distribution of time necessary to reach the nearest urban area, the remaining municipalities were classified in peri-urban, rural and isolated rural areas. For the present study the variable urbanization level of residence was dichotomized as follow: urban area - that includes urban and peri-urban areas - and rural area – that includes rural and very isolated rural areas.

Supplementary Tables

Table S1a. Crude odd ratios for delayed vaccination for HEXA-1 for prematurity classes (very preterm infants – VPTI; moderate and late preterm infants – MLPTI; full-term infants – FTI) and SGA status.

	HEXA-1		
	**Crude OR**	**95%CI**	**p**
**Gestational age at birth**			
FTI GA: ≥37 weeks	Ref.		
MLPTI GA: 32-36 weeks	1.32	1.20 - 1.45	<0.001
VPTI GA: <32 weeks	5.19	4.00 - 6.73	<0.001
**SGA status**			
≥10th percentile	Ref.		
<10th percentile	1.15	1.06 - 1.25	0.001

Table S1b. Adjusted odd ratios for delayed vaccination for HEXA-1 for prematurity classes (very preterm infants – VPTI; moderate and late preterm infants – MLPTI; full-term infants – FTI) and SGA status.

	HEXA-1		
	**Adjusted OR**	**95%CI**	**p**
**Gestational age at birth**			
FTI GA: ≥37 weeks	Ref.		
MLPTI GA: 32-36 weeks	1.19	1.07 - 1.32	0.001

VPTI GA: <32 weeks	4.63	3.50 - 6.14	<0.001
**SGA status**			
≥10th percentile	Ref.		
<10th percentile	1.12	1.03 - 1.22	0.01
**Sex**			
Male	Ref.		
Female	0.99	0.94 - 1.04	0.71
**Pregnancy outcome**			
Singleton	Ref.		
Multiple	1.31	1.13 - 1.50	<0.001
**Conceived by ART[1]**			
No	Ref.		
Yes	0.86	0.75 - 0.99	0.04
**Urbanization level of residence**			
Urban area	Ref.		
Rural area	1.10	1.03 - 1.16	0.002
**Birth hospital level**			
First level	Ref.		
Second level	1.38	1.31 - 1.45	<0.001
**Maternal nationality**			
Italian	Ref.		
Foreign nationality	1.03	0.96 - 1.10	0.40
**Maternal age at delivery**			
<25 years	Ref.		
25-34 years	0.91	0.82 - 1.01	0.07
>34 years	1.04	0.93 - 1.15	0.52
**Maternal education level**			
High school diploma or higher	Ref.		
Less than high-school diploma	0.94	0.88 - 1.00	0.04
**Maternal employment status**			
Employed	Ref.		
Unemployed	1.06	1.00 - 1.13	0.04
**Sibling birth order**			
Having older sibling	Ref.		
First born/only child	0.75	0.71 - 0.80	<0.001

[1] ART = assisted reproductive technologies.

Table S2a. Crude odd ratios for delayed vaccination for HEXA-3 for prematurity classes (very preterm infants – VPTI; moderate and late preterm infants – MLPTI; full-term infants – FTI) and SGA status.

	HEXA-3		
	**Crude OR**	**95%CI**	**p**
**Gestational age at birth**			
FTI GA: ≥37 weeks	Ref.		
MLPTI GA: 32-36 weeks	1.17	1.08 - 1.26	<0.001
VPTI GA: <32 weeks	1.80	1.44 - 2.22	<0.001
**SGA status**			
≥10th percentile	Ref.		
<10th percentile	1	0.94 - 1.08	0.79

Table S2b. Adjusted odd ratios for delayed vaccination for HEXA-3 for prematurity classes (very preterm infants – VPTI; moderate and late preterm infants – MLPTI; full-term infants – FTI) and SGA status.

	HEXA-3		
	**Adjusted OR**	**95%CI**	**p**
**Gestational age at birth**			
FTI GA: ≥37 weeks	Ref.		
MLPTI GA: 32-36 weeks	1.20	1.09 - 1.31	<0.001
VPTI GA: <32 weeks	1.82	1.44 - 2.31	<0.001

**SGA status**			
≥10th percentile	Ref.		
<10th percentile	1.00	0.93 - 1.07	0.96
**Sex**			
Male	Ref.		
Female	0.94	0.90 - 0.98	0.006
**Pregnancy outcome**			
Singleton	Ref.		
Multiple	1.03	0.91 - 1.17	0.61
**Conceived by ART**[1]			
No	Ref.		
Yes	0.90	0.80 - 1.01	0.08
**Urbanization level of residence**			
Urban area	Ref.		
Rural area	1.30	1.24 - 1.36	<0.001
**Birth hospital level**			
First level	Ref.		
Second level	1.23	1.18 - 1.29	<0.001
**Maternal nationality**			
Italian	Ref.		
Foreign nationality	1.01	0.96 - 1.07	0.60
**Maternal age at delivery**			
<25 years	Ref.		
25-34 years	0.93	0.85 - 1.01	0.11
>34 years	0.90	0.82 - 0.99	0.04
**Maternal education level**			
High school diploma or higher	Ref.		
Less than high-school diploma	1.02	0.97 - 1.08	0.44
**Maternal employment status**			
Employed	Ref.		
Unemployed	1.03	0.98 - 1.09	0.21
**Sibling birth order**			
Having older sibling	Ref.		
First born/only child	0.68	0.65 - 0.71	<0.001

[1] ART = assisted reproductive technologies.

Table S3a. Crude odd ratios for delayed vaccination for MMR for prematurity classes (very preterm infants – VPTI; moderate and late preterm infants – MLPTI; full-term infants – FTI) and SGA status.

	**MMR**		
	**Crude OR**	**95%CI**	**p**
**Gestational age at birth**			
FTI GA: ≥37 weeks	Ref.		
MLPTI GA: 32-36 weeks	1.03	0.95 - 1.11	0.001
VPTI GA: <32 weeks	1.51	1.20 - 1.92	<0.001
**SGA status**			
≥10th percentile	Ref.		
<10th percentile	1.01	0.94 - 1.08	0.76

Table S3b. Adjusted odd ratios for delayed vaccination for MMR for prematurity classes (very preterm infants – VPTI; moderate and late preterm infants – MLPTI; full-term infants – FTI) and SGA status.

	**MMR**		
	**Adjusted OR**	**95%CI**	**p**
**Gestational age at birth**			
FTI GA: ≥37 weeks	Ref.		
MLPTI GA: 32-36 weeks	1.04	0.95 - 1.14	0.36

VPTI GA: <32 weeks	1.62	1.25 - 2.10	<0.001
**SGA status**			
≥10th percentile	Ref.		
<10th percentile	1.00	0.93 - 1.08	0.94
**Sex**			
Male	Ref.		
Female	0.95	0.91 - 0.99	0.01
**Pregnancy outcome**			
Singleton	Ref.		
Multiple	1.12	0.99 - 1.28	0.07
**Conceived by ART[1]**			
No	Ref.		
Yes	0.83	0.74 - 0.92	<0.001
**Urbanization level of residence**			
Urban area	Ref.		
Rural area	0.91	0.87 - 0.96	<0.001
**Birth hospital level**			
First level	Ref.		
Second level	1.05	1.01 - 1.10	0.02
**Maternal nationality**			
Italian	Ref.		
Foreign nationality	0.82	0.78 - 0.87	<0.001
**Maternal age at delivery**			
<25 years	Ref.		
25-34 years	0.98	0.90 - 1.07	0.63
>34 years	0.95	0.87 - 1.04	0.29
**Maternal education level**			
High school diploma or higher	Ref.		
Less than high-school diploma	1.12	1.06 - 1.18	<0.001
**Maternal employment status**			
Employed	Ref.		
Unemployed	1.00	0.95 - 1.05	0.97
**Sibling birth order**			
Having older sibling	Ref.		
First born/only child	0.82	0.78 - 0.85	<0.001

[1] ART = assisted reproductive technologies.

Table S4a. Crude odd ratios for delayed vaccination for Var for prematurity classes (very preterm infants – VPTI; moderate and late preterm infants – MLPTI; full-term infants – FTI) and SGA status.

	Var		
	**Crude OR**	**95%CI**	**p**
**Gestational age at birth**			
FTI GA: ≥37 weeks	Ref.		
MLPTI GA: 32-36 weeks	1.06	0.97 - 1.15	0.17
VPTI GA: <32 weeks	1.69	1.32 - 2.18	<0.001
**SGA status**			
<10th percentile	Ref.		
≥10th percentile	1	0.94 - 1.08	0.79

Table S4b. Adjusted odd ratios for delayed vaccination for Var for prematurity classes (very preterm infants – VPTI; moderate and late preterm infants – MLPTI; full-term infants – FTI) and SGA status.

	Var		
	**Adjusted OR**	**95%CI**	**p**
**Gestational age at birth**			
FTI GA: ≥37 weeks	Ref.		
MLPTI GA: 32-36 weeks	1.07	0.97 - 1.17	0.17
VPTI GA: <32 weeks	1.78	1.35 - 2.35	<0.001

**SGA status**			
≥10th percentile	Ref.		
<10th percentile	1.00	0.93 - 1.08	0.99
**Sex**			
Male	Ref.		
Female	0.94	0.90 - 0.98	0.009
**Pregnancy outcome**			
Singleton	Ref.		
Multiple	1.13	0.99 - 1.29	0.07
**Conceived by ART[1]**			
No	Ref.		
Yes	0.79	0.71 - 0.89	<0.001
**Urbanization level of residence**			
Urban area	Ref.		
Rural area	0.93	0.88 - 0.97	0.003
**Birth hospital level**			
First level	Ref.		
Second level	1.05	1.00 - 1.09	0.04
**Maternal nationality**			
Italian	Ref.		
Foreign nationality	0.79	0.74 - 0.83	<0.001
**Maternal age at delivery**			
<25 years	Ref.		
25-34 years	0.99	0.91 - 1.08	0.90
>34 years	0.99	0.90 - 1.08	0.79
**Maternal education level**			
High school diploma or higher	Ref.		
Less than high-school diploma	1.09	1.04 - 1.15	0.001
**Maternal employment status**			
Employed	Ref.		
Unemployed	1.01	0.96 - 1.07	0.57
**Sibling birth order**			
Having older sibling	Ref.		
First born/only child	0.84	0.80 - 0.87	<0.001

[1] ART = assisted reproductive technologies.

Table S5a. Crude odd ratios for delayed vaccination for MenC for prematurity classes (very preterm infants – VPTI; moderate and late preterm infants – MLPTI; full-term infants – FTI) and SGA status.

	**MenC**		
	**Crude OR**	**95%CI**	**p**
**Gestational age at birth**			
FTI GA: ≥37 weeks	Ref.		
MLPTI GA: 32-36 weeks	1.07	0.97 - 1.19	0.16
VPTI GA: <32 weeks	1.66	1.30 - 2.12	<0.001
**SGA status**			
≥10th percentile	Ref.		
<10th percentile	1	0.92 - 1.10	0.89

Table S5b. Adjusted odd ratios for delayed vaccination for MenC for prematurity classes (very preterm infants – VPTI; moderate and late preterm infants – MLPTI; full-term infants – FTI) and SGA status.

	**MenC**		
	**Adjusted OR**	**95%CI**	**p**
**Gestational age at birth**			
FTI GA: ≥37 weeks	Ref.		
MLPTI GA: 32-36 weeks	1.06	0.95 - 1.19	0.28
VPTI GA: <32 weeks	1.89	1.45 - 2.46	<0.001

**SGA status**			
≥10th percentile	Ref.		
<10th percentile	0.99	0.90 - 1.08	0.78
**Sex**			
Male	Ref.		
Female	0.94	0.89 - 0.99	0.03
**Pregnancy outcome**			
Singleton	Ref.		
Multiple	1.26	1.08 - 1.47	0.003
**Conceived by ART[1]**			
No	Ref.		
Yes	0.81	0.69 - 0.95	0.009
**Urbanization level of residence**			
Urban area	Ref.		
Rural area	0.99	0.93 - 1.05	0.69
**Birth hospital level**			
First level	Ref.		
Second level	0.85	0.80 - 0.90	<0.001
**Maternal nationality**			
Italian	Ref.		
Foreign nationality	0.93	0.86 - 0.99	0.03
**Maternal age at delivery**			
<25 years	Ref.		
25-34 years	0.90	0.81 - 1.00	0.04
>34 years	0.91	0.81 - 1.02	0.10
**Maternal education level**			
High school diploma or higher	Ref.		
Less than high-school diploma	1.19	1.12 - 1.27	<0.001
**Maternal employment status**			
Employed	Ref.		
Unemployed	1.04	0.98 - 1.11	0.21
**Sibling birth order**			
Having older sibling	Ref.		
First born/only child	0.70	0.66 - 0.74	<0.001

[1] ART = assisted reproductive technologies.

Table S6a. Crude odd ratios for delayed vaccination for PCV-1 for prematurity classes (very preterm infants – VPTI; moderate and late preterm infants – MLPTI; full-term infants – FTI) and SGA status.

		**PCV-1**	
	**Crude OR**	**95%CI**	**p**
**Gestational age at birth**			
FTI GA: ≥37 weeks	Ref.		
MLPTI GA: 32-36 weeks	1.36	1.24 - 1.49	<0.001
VPTI GA: <32 weeks	5.62	4.34 - 7.29	<0.001
**SGA status**			
≥10th percentile	Ref.		
<10th percentile	0.29	1.08 - 1.27	<0.001

Table S6b. Adjusted odd ratios for delayed vaccination for PCV-1 for prematurity classes (very preterm infants – VPTI; moderate and late preterm infants – MLPTI; full-term infants – FTI) and SGA status.

		**PCV-1**	
	**Adjusted OR**	**95%CI**	**p**
**Gestational age at birth**			
FTI GA: ≥37 weeks	Ref.		
MLPTI GA: 32-36 weeks	1.21	1.09 - 1.35	<0.001

VPTI GA: <32 weeks	4.95	3.74 - 6.55	<0.001
**SGA status**			
≥10th percentile	Ref.		
<10th percentile	1.13	1.04 - 1.24	0.003
**Sex**			
Male	Ref.		
Female	0.98	0.93 - 1.04	0.55
**Pregnancy outcome**			
Singleton	Ref.		
Multiple	1.33	1.16 - 1.54	<0.001
**Conceived by ART[1]**			
No	Ref.		
Yes	0.90	0.78 - 1.03	0.14
**Urbanization level of residence**			
Urban area	Ref.		
Rural area	1.11	1.04 - 1.17	0.001
**Birth hospital level**			
First level	Ref.		
Second level	1.43	1.36 - 1.51	<0.001
**Maternal nationality**			
Italian	Ref.		
Foreign nationality	1.06	0.99 - 1.13	0.08
**Maternal age at delivery**			
<25 years	Ref.		
25-34 years	0.92	0.83 - 1.02	0.12
>34 years	1.04	0.93 - 1.16	0.48
**Maternal education level**			
High school diploma or higher	Ref.		
Less than high-school diploma	0.95	0.89 - 1.01	0.10
**Maternal employment status**			
Employed	Ref.		
Unemployed	1.07	0.99 - 1.12	0.07
**Sibling birth order**			
Having older sibling	Ref.		
First born/only child	0.76	0.72 - 0.80	<0.001

[1] ART = assisted reproductive technologies.

Table S7a. Crude odd ratios for delayed vaccination for PCV-3 for prematurity classes (very preterm infants – VPTI; moderate and late preterm infants – MLPTI; full-term infants – FTI) and SGA status.

	PCV-3		
	**Crude OR**	**95%CI**	**p**
**Gestational age at birth**			
FTI GA: ≥37 weeks	Ref.		
MLPTI GA: 32-36 weeks	1.13	1.04 - 1.22	0.003
VPTI GA: <32 weeks	1.39	1.11 - 1.73	0.004
**SGA status**			
≥10th percentile	Ref.		
<10th percentile	1	0.93 - 1.07	0.99

Table S7b. Adjusted odd ratios for delayed vaccination for PCV-3 for prematurity classes (very preterm infants – VPTI; moderate and late preterm infants – MLPTI; full-term infants – FTI) and SGA status.

	PCV-3		
	**Adjusted OR**	**95%CI**	**p**
**Gestational age at birth**			
FTI GA: ≥37 weeks	Ref.		
MLPTI GA: 32-36 weeks	1.15	1.05 - 1.26	0.004

VPTI GA: <32 weeks	1.35	1.06 - 1.72	0.01
**SGA status**			
≥10th percentile	Ref.		
<10th percentile	0.98	0.91 - 1.06	0.69
**Sex**			
Male	Ref.		
Female	0.93	0.89 - 0.97	0.002
**Pregnancy outcome**			
Singleton	Ref.		
Multiple	1.05	0.92 - 1.19	0.45
**Conceived by ART[1]**			
No	Ref.		
Yes	1.01	0.90 - 1.13	0.85
**Urbanization level of residence**			
Urban area	Ref.		
Rural area	1.36	1.29 - 1.43	<0.001
**Birth hospital level**			
First level	Ref.		
Second level	1.22	1.17 - 1.28	<0.001
**Maternal nationality**			
Italian	Ref.		
Foreign nationality	0.92	0.87 - 0.98	0.006
**Maternal age at delivery**			
<25 years	Ref.		
25-34 years	0.94	0.86 - 1.02	0.16
>34 years	0.91	0.83 - 0.10	0.05
**Maternal education level**			
High school diploma or higher	Ref.		
Less than high-school diploma	1.04	0.99 - 1.10	0.11
**Maternal employment status**			
Employed	Ref.		
Unemployed	0.99	0.94 - 1.04	0.79
**Sibling birth order**			
Having older sibling	Ref.		
First born/only child	0.72	0.69 - 0.76	<0.001

[1] ART = assisted reproductive technologies.
